# Trusted health system implementation strategies to increase vaccination (TRUE SYNERGI): a stepped-wedge cluster randomized trial to reduce HPV-related cancers

**DOI:** 10.1186/s12889-025-22273-7

**Published:** 2025-04-09

**Authors:** Daisy Y. Morales-Campos, Prajakta Adsul, Yuanyuan Liang, Erin Donovan, Leticia R. Moczygemba, Jessica A. Kahn

**Affiliations:** 1 Department of Health Promotion and Behavioral Sciences, Center for Health Promotion and Prevention Research, The University of Texas Health Science Center at Houston, Houston, TX USA; 2 Division of Epidemiology, Biostatistics, and Preventive Medicine, Department of Internal Medicine, School of Medicine, Comprehensive Cancer Center, Cancer Control and Population Sciences Research Program, Comprehensive Cancer Center; University of New Mexico, Albuquerque, NM USA; 3 Division of Biostatistics and Bioinformatics, Department of Epidemiology and Public Health, School of Medicine, University of Maryland, Baltimore, MD USA; 4 Moody College of Communication, The University of Texas at Austin, Austin, TX USA; 5 College of Pharmacy-Health Outcomes, The University of Texas at Austin, Austin, TX USA; 6 Senior Associate Dean for Clinical and Translational Research, Albert Einstein College of Medicine, Bronx, NY USA

**Keywords:** HPV vaccination, Latino/a, Implementation, Sustainability, Hybrid type 2 study, Stepped-wedge cluster randomized trial, Federally Qualified Health Centers

## Abstract

**Background:**

Despite the availability of highly effective HPV vaccines that can reduce HPV-associated cancer mortality, HPV vaccination rates in Texas rank 48th nationwide. Although evidence shows Latino parents are more accepting of HPV vaccination than non-Hispanic parents, this disparity in vaccination rates underscores the importance of understanding Latino parental HPV vaccine hesitancy. Latinos/as typically receive healthcare at Federally Qualified Health Centers (FQHCs), which often need support implementing and improving access to evidence based preventive services. However, the current literature around implementation comes from large integrated healthcare systems and there is limited research around what works in the FQHC settings with Latino/a patients. Preliminary data from our previous work suggest practice facilitation is a feasible approach for building the capacity in FQHCs to select and implement provider- and practice-level strategies for increasing vaccination rates.

**Methods:**

This proposal considers the HPV vaccine as the evidence-based intervention and describes the rational and study design for “**TRU**sted h**E**alth **SY**stem implementatio**N** strat**EGI**es to increase vaccination (TRUE SYNERGI)”, a hybrid type 2 study that uses previously-piloted implementation strategies (i.e., practice facilitation, provider education, among others) to influence provider recommendations (implementation outcome) and practice-level vaccination rates (effectiveness outcome). To test whether these facilitator-driven implementation strategies influence our implementation and effectiveness outcomes, we will use a stepped-wedge cluster randomized trial and randomize three FQHCs (*n* = 9 practices, 3 per FQHC) to three clusters. We will conduct baseline assessments at each practice, which will provide data to assist the practice facilitator in engaging with the providers and leadership to develop a tailored implementation plan for each practice. In addition, we will employ theory-guided, qualitative methods, to assess the complexity associated with context and the recipients involved in the implementation of strategies in practices, along with sustainability.

**Discussion:**

The study will advance our understanding of what it means to conduct implementation research in resource limited practices that work with populations experiencing substantial disparities. Findings from the current study will inform national implementation efforts and contribute towards future research targeting dissemination and scale-up, key foci for health equity focused implementation research.

**Trial registration:**

Registered in ClinicalTrials.gov (NCT06598475) on September 9, 2024.

**Supplementary Information:**

The online version contains supplementary material available at 10.1186/s12889-025-22273-7.

## Introduction

Approximately 46,143 new cases of human papillomavirus (HPV) associated cancers occur annually in the U.S. among women and men [1]. Persistent HPV infection causes approximately 90% of anal cancers, 70% of oropharyngeal cancers, and 60-70% of vaginal, vulvar, and penile cancers; the most common is cervical cancer [2]. Significant racial, ethnic, and socioeconomic disparities in cervical cancer incidence and mortality exist, and disparities vary by geographic region [3]. For example, Hispanic women in Texas, compared to those nationwide, experience higher cervical cancer incidence (12 vs. 10/100,000) and mortality (3 vs. 2/100,000) [4–7]. Primary prevention through HPV vaccination and secondary prevention through cervical cancer screening are two known evidence-based interventions that reduce HPV infection and HPV-associated pre-cancers and cancers [8, 9]. The U.S. Advisory Committee on Immunization Practices recommends routine HPV vaccination for 11–12 year olds with catch-up vaccination to age 26 years [10, 11]. Despite these recommendations and widespread availability of HPV vaccines [12], vaccine uptake is suboptimal [13, 14].

Latino/a parents are the primary decision-makers in their child receiving the HPV vaccine and are more likely to report willingness to vaccinate if they have higher knowledge levels, believe the vaccine prevents cervical cancer and is safe, and perceive few barriers to accessing the vaccine [15–18]. Despite evidence showing Latino parents are more accepting of HPV vaccination than non-Hispanic parents [19], vaccination rates are still low. In Texas, HPV vaccination rates rank 48th nationwide [20]. Texas Latino/a 13–17 year-olds compared to U.S. Latino/a adolescents had lower HPV vaccine completion rates (45% vs. 54%) [21] but similar rates for meningococcal and tetanus, diphtheria, and pertussis vaccines, underscoring the importance of understanding Latino parental HPV vaccine hesitancy to address this disparity [22].

Factors associated with the suboptimal uptake of vaccinations are multilevel, including at the individual (parent and the adolescent)-, provider-, and the clinical practice-levels [23]. In addition to inadequate access to the vaccine, parents and adolescents may encounter individual barriers and system-level barriers [24, 25]. Vaccine hesitancy is another major individual factor underlying suboptimal uptake and drivers include media misinformation, lack of knowledge and awareness about HPV and HPV vaccines, belief that HPV vaccines are not necessary, concerns about adolescents initiating or increasing sexual activity, parental concerns about vaccine effectiveness and safety, and lack of strong provider recommendation [26]. Additionally, providers face challenges in administering multiple vaccine doses [27]; parental misconceptions [28], parental religious objections [29]; and beliefs about the appropriate timing of the vaccine [30]. Clinical practice-level barriers including inconsistent healthcare visits [31] by adolescents and missed opportunities at acute/chronic disease management visits [32, 33] also contribute to low vaccination rates.

Among all of these factors, the strongest predictor of vaccine initiation and completion rates is a parent receiving a healthcare provider’s (HCP) recommendation (provider-level) [30, 34]. Previous research shows that Latino/a parents value information and HPV vaccine recommendations from their child’s HCP [19, 35, 36]. However, low-income and racial/ethnic minority adolescents were less likely than their high-income or white peers to report HCPs recommending the vaccine to them and completing the series [37]. HCPs serving urban, low-income, mixed race/ethnic adolescents indicate that lack of awareness and knowledge of the vaccine [38], negative attitudes towards vaccination, anticipated parental resistance [39], and lack of provider time with patients [31, 40], contribute to their lack of strong and/or consistent recommendations for HPV vaccines.

Recommendations from the Community Preventive Services Task Force [41] (Task Force) and prior research [42–44] demonstrates that interventions addressing more than one level (individual, provider, and practice) have the greatest effect on increasing vaccination rates. Currently, the Task Force recommends the following evidence-based implementation strategies to increase vaccination rates: provider assessment and feedback, provider reminders, patient reminder/recall, standing orders, and practice-based patient education [41]. At the provider-level, previous research has demonstrated effectiveness when providers are trained to use patient education materials [45, 46], distribute patient education materials [47], communicate with parents [45], and connect to online resources [45]. Other studies have focused on the practice-level by using audit and feedback [48–50], and/or electronic decision support tools or provider reminders [48, 51]. From HPV vaccine intervention research, we know that (1) working with healthcare providers, (2) focusing on implementation on multiple levels, and (3) using theory in intervention development are best practices, but in a recent systematic review few interventions were multilevel and employed theory [52]. The setting for the majority of these research studies were large, urban, hospital-based primary care practices [48, 51, 53], whereas few were conducted in Federally Qualified Health Centers [54–56] (FQHCs). Research also demonstrated that strategies tailored to specific cultural contexts were effective in promoting health behavior change [57, 58] but few incorporate Latino/a patients or parents [45, 47]. Implementation studies to increase vaccination in resource-limited settings like FQHCs [59–61], especially those serving low-income, uninsured/underinsured, and racial/ethnic minority populations, are essential to make progress towards the Healthy People 2030 target of 80% for HPV vaccination [62].

To address these gaps, our study team pilot tested TRUE SYNERGI in two Texas FQHCs, serving predominantly Latino/a adolescents and located in areas with high cervical cancer incidence rates for Latinas. Combining provider- and practice-level strategies using practice facilitation [63, 64], over a two-year implementation period, HPV vaccine initiation more than quadrupled (from 4 to 19%) in FQHC A and increased from 45 to 66% in FQHC B [65]. These preliminary data suggest external facilitation is a feasible approach for building the capacity in predominantly Latino serving FQHCs to select and implement provider- and practice-level strategies aimed at increasing vaccination rates. The study described in this manuscript builds on the pilot and aims to: 1) assess patient/parent-, provider- and practice-level characteristics (including vaccine hesitancy) that may influence the selection of implementation strategies, 2) use a participatory approach led by an external facilitator in each clinical practice to implement strategies, and 3) evaluate the implementation and identify factors that could influence future sustainability. We expect study findings to advance our understanding of implementation research in resource-limited practices that serve populations experiencing substantial health disparities, and to identify strategies that improve delivery and increase vaccine uptake. These findings could be disseminated to FQHCs nationwide that may share similar challenges in providing healthcare services to these patient populations and inform a future proposal focused on sustainability, an emerging area for equity focused implementation research.

## Methods

### Overall design

The purpose of TRUE SYNERGI is to test the implementation and effectiveness of provider- and practice-level strategies aimed at increasing vaccination rates, first in a larger sample of practices and second, to improve our understanding of what combination of strategies influence provider recommendations and practice-level vaccination rates and under what conditions. We use a hybrid type 2 study design [66], focusing on understanding implementation aspects of an HPV vaccination focused set of strategies in a FQHC setting [66–68]. We are using a stepped-wedge, cluster randomized trial, among three predominantly Latino serving FQHCs with nine practice sites in Texas. We selected this design due to logistical, practical, and financial constraints in FQHC settings that make it more feasible for implementation to take place in stages [69] (See Table 1).

**Table 1:**
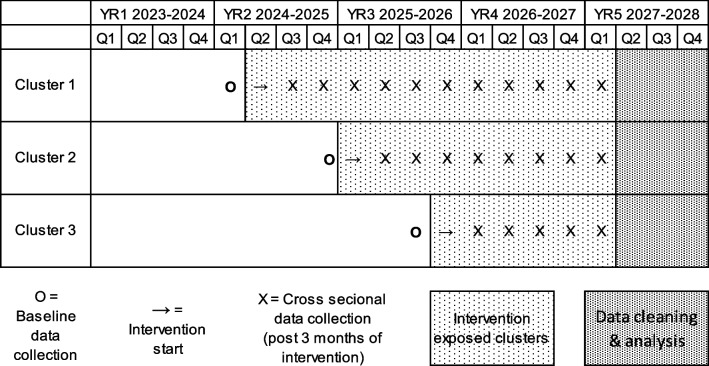
Research design

### Setting and randomization

Our three FQHC collaborators are Community Health Network (CHN), Project Vida Health Center (PVHC), and Spring Branch Community Health Center (SBCHC), which service different areas of Texas. We recruited these FQHCs in collaboration with through the Texas Association of Community Health Centers (TACHC). The Director of Clinical Affairs emailed their members a description of the study, the inclusion criteria below, and the PI’s email to contact directly if interested in participating. *Inclusion Criteria*. FQHCs were eligible for the trial if they met the following criteria: (1) have less than 60% HPV vaccine initiation rate for 11–12 year old adolescents overall at their practice sites (primary outcome); (2) have family medicine and/or pediatric practices; and (3) have a total adolescent patient population at least 50% Latino/a. *Exclusion Criteria*. FQHCs were excluded if they participated in the pilot study. *Randomization*. Our biostatistician will randomly assign a FQHC out of the three participating to begin using a simple random sorting. Once randomized, the FQHC will stay in their assigned position all five years.

### Conceptual framework

**Fig. 1:**
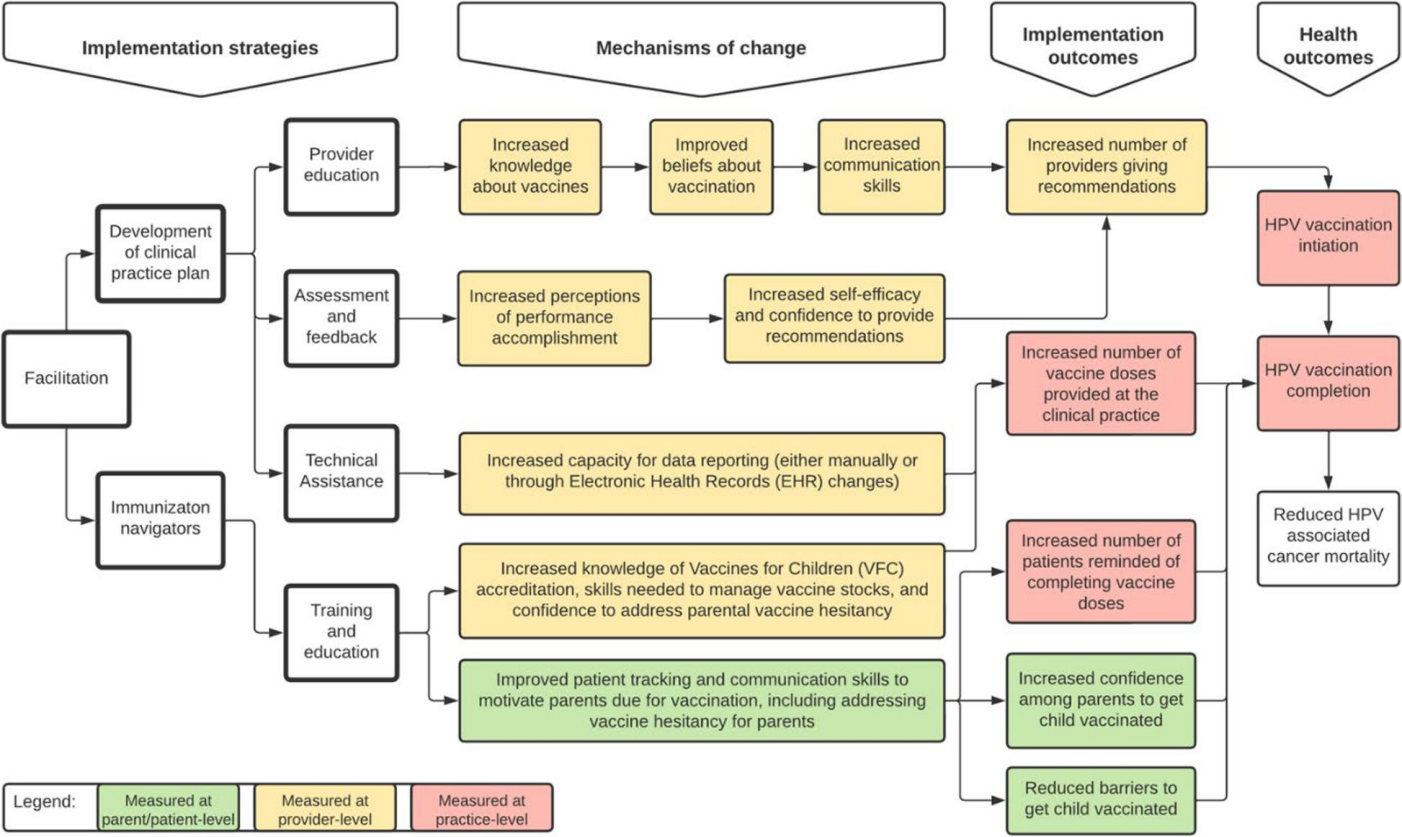
Conceptual framework

Figure 1 shows the conceptual framework for this study [70, 71].Given the feasibility shown by TRUE SYNERGI, the external facilitator will use a participatory approach to implement these strategies: provider education, assessment and feedback, technical assistance, training and education for the clinical staff, immunization navigator, and development of the clinical practice plan. We will examine the mechanisms by which these strategies influence the implementation outcomes at the level of the parent/patient, provider, and clinical practice using both qualitative and quantitative methods. Given this is an implementation trial, we will measure both effectiveness (i.e., HPV vaccination initiation and completion among 11–12 year olds and among 13–17 year olds) and implementation outcomes (i.e., number of providers giving HPV vaccine recommendations, number of vaccine doses provided, number of patient reminders/recalls made for vaccine completion, and confidence among parents in vaccinating their children).

### Pilot study and implementation collaborators

In the pilot study, we emphasized community building by developing rapport and trust between the research team (including external facilitator) and FQHC leadership, HCP, and staff through meet and greets, monthly planning meetings with each implementation team, and quarterly partner meetings with study collaborators. Laying this foundation with our FQHC partners was essential for the research team in using practice facilitation to identify contextual challenges in practices and combine provider- and practice-level strategies to develop a clinical practice plan and enact changes. A team consisted of: FQHC administrators, HCPs, immunization navigators, and information technology specialist. Practice facilitation involved using an external facilitator with a sound understanding of the strategies to be implemented, the team that would enact change in the practice [63]. The external facilitator had a background in public health interventions, a medical degree, and experience communicating with HCPs. First, we conducted a baseline assessment per practice, then presented a summary report (i.e., vaccine coverage, provider performance, and practice characteristics) to the team. Second, the external facilitator, using a participatory approach and action learning techniques, worked with the team to select specific implementation strategies and encouraged them to reflect and think systematically about how to improve their practice based on best available evidence, providing training on robust methods [64]. Third, after tailoring the clinical practice plan to the practice’s environment, the team worked with the external facilitator to enable its adoption through monthly meetings to provide technical assistance, support problem solving, and monitor progress. We will use these steps from the pilot study in our current study. *Each FQHC will participate in three components of TRUE SYNERGI as part of the RCT: (1) baseline assessments, (2) implementation of facilitator-led implementation strategies, and (3) evaluation of factors influencing sustainability of facilitator-led implementation strategies.* Table 2 shows our community-academic collaborators and their roles in the RCT.

**Table 2:** TRUE SYNERGI implementation collaborators and their roles

Collaborators	Program Tasks, Roles and Responsibilities
The University of Texas Health Science Center at Houston	Provide overall program direction, leadership, and oversight; oversee personnel, budgeting, evaluation, and reporting; and oversee the planning, implementation, and evaluation of the multilevel intervention.
Latino Research Institute at The University of Texas at Austin	Data management; guidance and implementation of data quality control procedures; and data analyses and interpretation.
The University of New Mexico Comprehensive Cancer Center	Inform the development of the study methods and measures, as they relate to the implementation of strategies/interventions and outcomes; provide guidance and feedback when needed, on data collection activities and preliminary data interpretation;and participate in data analysis, data interpretation, and help develop a manuscript on the project’s pre-implementation work.
Albert Einstein College of Medicine	Provide expertise and guidance on design of survey instruments, design of interventions, development of qualitative and quantitative research methods, statistical analysis, and manuscript development.
The University of Maryland Baltimore	Provide guidance on study design, randomization procedures, measurement protocol development and database creation; work with study team to ensure data management and data quality control procedures are in place and conduct data analyses appropriately; and assist in data interpretation and manuscript preparation and dissemination.
Texas Association of Community Health Centers	Provide guidance with program planning; delivery of provider education/training and booster trainings; delivery of immunization champion training and booster trainings; and provide guidance on program evaluation and technical assistance activities.
San Antonio AIDS Foundation	Train and support the external facilitator in training of the practices’ immunization navigators and baseline assessments; provide all training materials and corresponding learning assessment tools on the use and implementation of clinic-based, evidence-based interventions; and train the external facilitator in the use of these training materials and tools.
Community Health Network, Project Vida Health Center, Spring Branch Community Health Center	Coordinate efforts across the three practice sites in the research study; support and honor the process of identification of eligible parents of adolescent patients to participate in surveys; oversee the planning and implementation of assessments, provider education, immunization navigator training, and clinical plan development; facilitate collection of data needed for assessments, trainings, and clinical plan development in coordination with UTHealth Health research staff; supervise the immunization navigators and coordinate efforts of the external facilitator with the practices (i.e., scheduling trainings, monthly technical assistance meetings, and EHR HPV vaccination rate reports); and report project progress to UTHealth Houston research team regularly.

### RCT provider and practice-level implementation strategies

The facilitator-driven implementation strategies used to increase HPV vaccine uptake in rural FQHC practices [29, 65] include development and implementation of a clinical practice plan, provider education, assessment and feedback plus technical assistance, and immunization navigator training and education.

#### Development and implementation of the clinical practice plan

This strategy consists of facilitator-led HCP education, assessment and feedback, and technical assistance. The external facilitator will conduct six modules over three months. HCP will complete a brief evaluation to receive continuing education units and evaluate the overall program after each module. In modules 5–6, the external facilitator will assist the implementation team in creating a clinical practice plan (or plan), and afterwards provide technical assistance plus assessment and feedback during its implementation. The external facilitator will also train immunization navigators to implement the plan, and conduct booster trainings and site visits every six months, to ensure performance does not deteriorate.

#### Immunization navigators

Navigators help guide patients and their families to overcome barriers to immunization by supporting, educating, and assisting families navigate the healthcare system [72]. We will leverage existing personnel and determine who is most appropriate to fulfill this role. The external facilitator will train immunization navigators to implement the plan.

### Recruitment, sample size, and retention plan

**Fig. 2:**
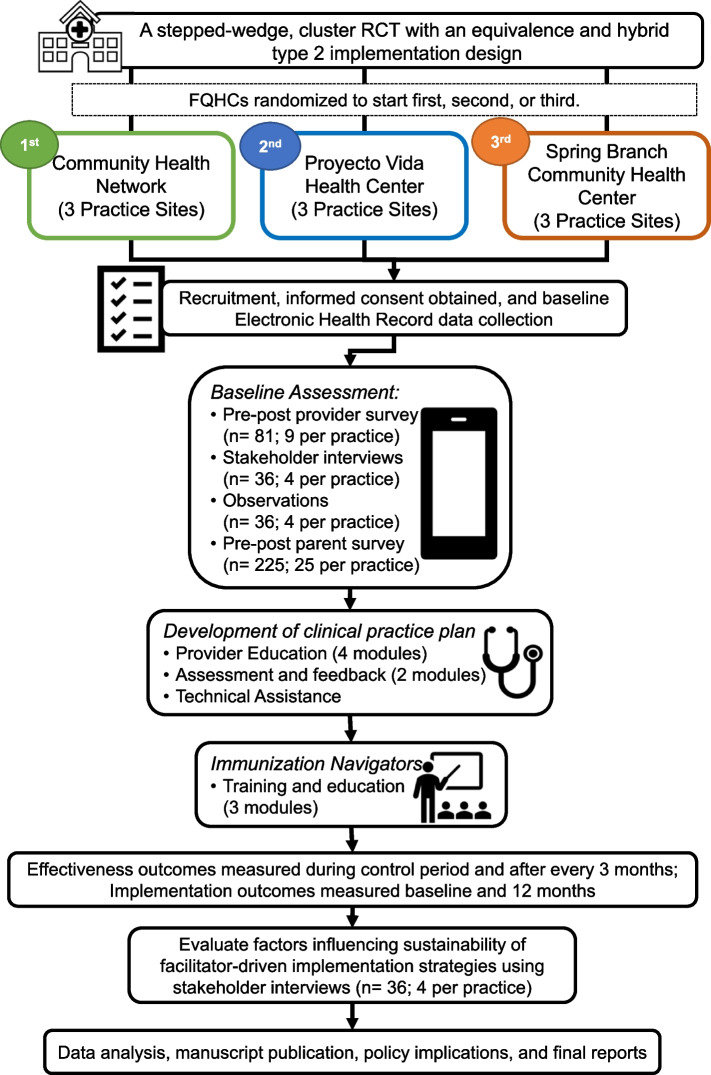
Federally Qualified Health Center (FQHC) and participant timeline

In Year 1, the academic research team and FQHC leadership will engage with practice sites to introduce practice coordinators, HCP, and staff to the study and establish a memorandum of understanding (MOU) outlining what study participation involves. Once FQHC leadership sign the MOU, we will randomize the FQHC. Participating HCP (*n* = 36; 4 per practice), staff members (*n* = 45; 5 per practice), implementation team (*n* = 36; 4 members per practice), and parents of patients aged 11–17 (*n* = 225; 25 per practice). HCP (*n* = 36; 4 per practice) and staff identified to serve as Immunization Navigators will receive the training and education. We will also conduct semi-structured interviews with the implementation team from each practice setting (*n* = 36) to identify key factors that influence sustainability of the strategies twelve months after implementation. We will only collect electronic health record (EHR) data from a sample of patients aged 11–12 and 13–17 based [73]. Our outcome data will be single measurements taken from individual patients, but we will randomly select patient EHR data for each data pull (cross-sectional data). See Fig. 2 for participant timeline.

We will test whether facilitator-driven provider- and practice-level implementation strategies increase provider recommendations and vaccination rates in three practices per FQHC. To ensure retention of the FQHCs and their staff during the trial, our project emphasizes active engagement using a participatory approach of the HCP and staff at each practice to increase capacity, assess barriers and gaps in vaccination services, and develop clinical practice plans. We have also integrated into our project plan continual assessment and feedback on performance indicators to provide ongoing quality control for participating practices. To ensure the sustainability of the program beyond funding, we will enhance the capacity of the FQHCs by providing trainings, technical assistance with implementing and adopting clinical practice plans, and developing new tools (e.g., provider reminders, patient reminders/recalls) to integrate into their existing practice systems. The multi-faceted approach described in our project plan has the potential to result in the adoption of provider- and practice-level evidence‐based strategies in practices, which will improve HPV vaccination (initiation and completion) rates among their adolescent patients.

### 1. Baseline assessments

The research team will conduct a baseline assessment using multiple data collection methods with the implementation team to identify practice- and provider-level characteristics that influence HPV vaccination. We will use these data to guide the selection of specific implementation strategies. Data collection and analysis will occur separately for each method and we triangulate and organize these data, using the integrated-Promoting Action on Research Implementation in Health Services (i-PARIHS) framework [63] to provide a comprehensive contextual assessment.

### Data collection

We will conduct the following assessments at each practice site:

- *HCP and staff surveys*. The FQHC’s designee will e‐mail an invitation with an embedded link to all providers and staff at each practice site to participate in a brief survey [74–77]. The survey measures HPV vaccine knowledge, attitudes, provider confidence responding to vaccine hesitant parents, and both provider-and practice-level behaviors regarding HPV vaccination.
- *Implementation Team Remote Interviews*. The FQHC’s designee will recruit the practice implementation team and encourage participation. The external facilitator will conduct interviews focusing on conducting a practice readiness assessment [78], facilitating a discussion around their practice level data, and identifying challenges to achieving target vaccination rates.
- *Observations*. Research staff will conduct observations of patient flow and clinical workflows to determine how to best implement strategies in the practice setting. Research staff will take detailed notes of what occurs during the visit, how a patient is identified as needing the HPV vaccine, and the process the patient goes through for receiving (or not receiving) the vaccine.
- *Parent surveys*. Research staff will conduct a cross-sectional, self‐administered, REDCap survey evaluating parents’ HPV vaccine knowledge, behaviors, general and HPV related vaccine hesitancy, self-efficacy in and reducing perceived barriers to vaccinating their child, experience with provider’s HPV vaccine recommendation, and practice service [35, 79–83], Practice staff will invite parents of children ages 11–17 who are scheduled for well-child check during the baseline and post assessment periods and *have not received the HPV vaccine* to participate.

### Analyses

Data collection and analysis will occur separately for each method, and we will triangulate and organize these data, using i-PARIHS. For the HCP/staff and parent surveys, research staff will use simple univariate and bi‐variate analyses to summarize the data in SPSS. We will utilize a content analysis [84, 85] approach for implementation team interview data. We will develop themes using an iterative process where analysts use conceptual labels (or codes) to categorize discrete concepts. Analysts will independently code transcripts and then meet to discuss emerging themes, discussing disputed sections until reaching consensus about coding categories. Based on observation notes, research staff will create a flowchart representing how the practice operates, the amount of time patients spend at any given point during their visit, and who the patient encounters. Guided by i-PARIHS’ *Outer Setting (e.g.,* participant needs and resources, external policies and incentives) and *Inner Setting (e.g.,* readiness for implementation, leadership engagement, available resources) contextual domains, we will triangulate the data and create summary reports.

### 2. Implementation of facilitator-led implementation strategies

Implementation strategies are methods or approaches that promote implementation indicators or outcome [86]. We will be focusing our efforts on the provider- and practice-level strategies described above. We will document the facilitation-driven implementation strategies selected by the clinical practice in their clinical practice plan. The i-PARIHS framework [63] will guide our understanding of the implementation of each of these strategies in the FQHCs. We will utilize the i-PARHS framework to guide the monthly meetings that will provide technical assistance if needed, assist in problem solving, and conduct a check-in, during the implementation period (defined as the first year after the start of project activities). Observational data in the form of monthly meeting notes and external facilitator debriefs will provide an in-depth understanding of the complex and dynamic *context* and the *recipients* (e.g., providers, immunization navigators, FQHC leadership) involved in the implementation at the practice settings. Baseline assessment findings will provide an in-depth understanding of the *context* and the *recipients*, which will enable the external facilitator to promote tailored implementation of strategies during implementation. We will utilize the i-PARIHS framework to review progress pre- and post-implementation and evaluate how strategies influenced implementation.

### Data collection

Our approach uses a stepped-wedge, cluster randomized control design to test the strategies used by the practice [87]. We will randomize three FQHC’s with three practices each (*n* = 9 practices), who meet eligibility criteria requirements, to begin the intervention first, second, or third. All practices within a given FQHC are in the same cluster. The design involves random and sequential crossover of an FQHC’s 3 practices from the control period to implementation of the facilitator-driven strategies until all are exposed [87]. We will assess primary and secondary effectiveness outcomes at baseline and every three months afterward. We powered the trial based on the primary outcome of HPV vaccine initiation and completion of 11–12 year olds.

#### Measurement and evaluation

We will assess primary and secondary effectiveness outcomes during the control period and after every 3 months, using de-identified, randomly selected, EHR data from patients aged 11–12 and 13–17 per practice. We will incorporate the general guidelines of the Immunization Quality Improvement for Providers protocol developed by the CDC [73]. The protocol recommends collecting 50 EHRs for every 150 patients. A trained information technology specialist, who is employed at each site (PVHC, CHN, and SBCHC), will provide de-identified, randomly selected, EHR data to the data manager. The external facilitator will be responsible for evaluation activities related to HCP, immunization navigators, and practice staff. Immunization navigators will enter and store process evaluation data onto REDCap. We will use these data to evaluate the clinical practice plan and track immunization uptake and dose compliance through the EHR. Implementation outcomes will be assessed using pre-post surveys in the baseline assessment and EHR reports. We will use observational data in the form of monthly meeting notes and external facilitator debriefs to assess the complexity associated with *context* and the *recipients* involved in the implementation at the practice settings as they influence implementation.

#### Effectiveness outcomes

The *primary outcome* is timely HPV vaccine initiation and completion (2 doses) rates of 11-12 year olds. We define completion as a second valid dose 6–12 months after their first dose. *Secondary outcomes* include timely initiation and completion (2 or 3 doses) for adolescent patients 13-17 years old (catch-up). For 13-14 year olds, we define completion as valid dose 6–12 months after their first dose. For 15–17 year olds, we define completion as a second valid dose 1-2 months after their first dose and a third valid dose 6 months after their first dose. Our outcome data will be single measurements taken from individual patients, but we will randomly select patient EHR data for each data pull [87]. These patient data will also include demographic data (race/ethnicity, gender, and age), which we will use to facilitate statistical analyses where potential confounding by these variables should be controlled.

#### Implementation outcomes

We will measure the implementation outcomes at the level of the provider (increase the number of providers giving HPV vaccine recommendations and their confidence in recommending the vaccine to hesitant parents), practice (increase in the number vaccine doses provided per practice and increase in the number of reminders provided to patients for HPV vaccine dose completion), and at the level of the patient/parent (increase parental confidence and reduce perceived barriers in vaccinating their children). We will conduct the HCP/staff and parent surveys 12 months after project activities start. In addition, we will also use the i-PARIHS framework [63] to gather observational data during the implementation period, which will enable us to track and review the progress of each practice.

### Analyses

#### Sample size justification

Based on the 2019-2021 data from three participating FQHCs, the average number of 11-12 year old patients seen per practice is 109 per 3 months, the HPV initiation rate ranges from 24.6% to 40.9% with an ICC of 0.026, and the HPV completion rate ranges from < 1% to 14.7% with an ICC of 0.003. Conservatively, we assume there will be an average of 30, 11–12 year old patients per practice per 3 months. We assume the average HPV initiation rate during the control period varies from 20 to 60% with an ICC of 0.026, and the average HPV completion rate during the control period varies from 3% to 40% with an ICC of 0.003.

A sample of 9 practices (three practices per FQHC) in a custom stepped-wedge design (see Table 3, 0 means control period, “.” means implementation/transition period, and 1 means intervention maintenance period) with 15, 3-month time periods, and an average of 420(= 30 × (15–1)), 11–12 year old subjects per practice with an average of 30 subjects per practice per 3-month (for a total sample size of 30 × (15–1) × 9 = 3,780 subjects) achieves 80% power to detect a minimum difference of 0.094 to 0.118 in initiation rate between the intervention maintenance period (depending on the initiation rate in the control period, see Table 4) and the control period using a two-sided Wald Z-Test, an ICC of 0.026, and a significance level of 0.050 (PASS version 2022). This study design achieves 80% power to detect a minimum difference of 0.039 to 0.112 in the HPV completion rate between intervention maintenance and control periods depending on the completion rate in the control period and the value of ICC of 0.003 (Table 4).

**Table 3:**
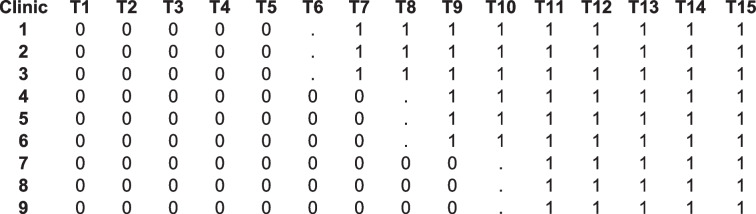
Custom stepwise design matrix plot

**Table 4:** Minimum detectable difference at 80% power using proposed design

Outcome	Intra-cluster correlation coefficient (ICC)	Rate in the control period	Minimum detectable difference at 80% power	Minimum detectable rate in the intervention period
HPV Initiation	0.026	0.2	0.094	0.294
		0.3	0.108	0.408
		0.4	0.116	0.516
		0.5	0.118	0.618
		0.6	0.116	0.716
HPV Completion	0.003	0.03	0.039	0.069
		0.05	0.05	0.1
		0.1	0.069	0.169
		0.15	0.082	0.232
		0.2	0.092	0.292
		0.3	0.105	0.405
		0.4	0.112	0.512

#### Statistical analysis plan

The same analytic strategy will be applied to each age group (11–12 vs. 13–17) for each HPV outcome separately. Both patient level characteristics (e.g., gender and ethnicity) and practice level characteristics (e.g., practice location and size) will be summarized using descriptive statistics and compared across the three FQHC groups using ANOVA or Kruskal-Wallis test for continuous variables and Chi-square or Fisher’s exact test for categorical variables. To compare the effect of facilitator-driven implementation strategies during the exposure period with the control period on the binary HPV outcomes, generalized linear mixed models (GLMMs) will be used with fixed effects for time and intervention as well as a random effect for practice, while considering patient-level and practice-level characteristics as needed. Heterogeneity of intervention effects will be examined by testing the interaction terms between intervention and potential effect.

#### Observational data analysis plan

We will employ a rapid content analysis technique to extract the most salient information regarding the context and the recipients from these data [88]. The method is widely used in health services research, and has recently been used in implementation research to maintain rigor and produce a quick turnaround of results. As described earlier, we will follow a systematic process of examining and categorizing data. We will organize the emerging code under the domains of innovation, recipients, or context (informed by i-PARIHS) as they relate to implementation at each practice site and across practice sites.

### 3. Evaluating sustainability of facilitator-led implementation strategies

Since sustainability of public health interventions in FQHCs is a persistent challenge, we examine key contextual factors and explore their relationship with sustainability of strategies, using the Integrated Sustainability Framework (ISF) [89]. Sustainability is conceptualized as a dynamic and iterative process over time that is influenced by multiple determinants at the different socioecological levels [89, 90]. This framework lists factors relevant to clinical settings, categorized under the domains of *outer context* (i.e., sociopolitical context, leadership, funding, etc.), *inner context* (i.e., staffing, navigators, etc.), *intervention characteristics* (i.e., adaptability, fit, etc.), *implementation processes* (i.e., partnerships, trainings, etc.), and *implementer characteristics* (i.e., related to the provider). We will use the observational data collected during the implementation period to identify the factors that influenced implementation and use these as a base to further query about sustainability in FQHCs.

### Data collection

Twelve months after implementation, we will also conduct semi-structured interviews with implementation team to identify key factors that influence sustainability of the strategies over the next three-five years. We will use interviews to gain in-depth insight and explore the real-world complexity associated with sustaining interventions in FQHCs, as recommended in previous literature [89, 90]. Informed by the ISF [89] and the observational data collected during the implementation period, we will work with our study team to develop an interview guide consisting of open-ended questions. Similar data collection procedures will be followed as described in baseline assessment for implementation team interviews.

### Analyses

Data analyses will follow a systematic process of examining, categorizing and tabulating the data from the transcripts to answer the research questions [85]. The analysts will read the interview transcripts separately to look for concepts and ideas described by the participants. Based on the interview questions and the initial concepts, a codebook will be created consisting of codes and their definition. Data interpretation will be based on: actual words used by the respondents, frequency, extensiveness and intensity of concepts, and the larger trends that emerge from an accumulation of evidence [91]. We will create descriptive statements that summarize these codes under the specific domains as described in the ISF [89] and organize these in a matrix format. We will use quotes to highlight comparisons both within and between transcripts. To report the findings from the qualitative inquiry, a conceptually ordered display [92] will be created to showcase the relationships between the concepts to explain sustainability of the strategies and implementation outcomes in the FQHC context, across settings.

### Data management and confidentiality

The research team trained in human subjects research and ethics will protect participants' privacy and confidentiality by 1) removing personally identifiable information from study records, 2) assigning a unique numerical identifier to each participant for their records, and 3) conducting interviews in a closed office space to keep any private information learned during interviews confidential. The research coordinator will securely store paper records in a locked filing cabinet. The data manager will store electronic records on a password-protected database and back up on a secured server. The PI will retain study data that do not contain personally-identifying information indefinitely to allow for review and reanalysis of data. Record retention and destruction of identifiable data will take place in compliance with NIH policies governing record maintenance, retention, and applicable regulations.

### Data and safety monitoring plan

The Pl will monitor data and safety on a continual basis. Annual reports will be submitted to The University of Texas Health Science Center at Houston (UTHealth Houston), Institutional Review Board (IRB) and National Institutes of Health (NIH). An Independent Monitor for the proposed study will be designated to semi-annually review study accrual, adverse events, and preliminary findings (process and outcome) as they become available. We will use this information to make any necessary modifications to the study. All adverse events will be reported immediately upon hearing the UTHealth Houston, IRB and to our collaborating FQHC partners. This intervention is believed to have very few risks for participants, and we anticipate very few to no study-related adverse events. If adverse events are reported that appear study-related, severe, and in excess to that expected, they will be reported to National Institutes of Health.

### Ethics and dissemination

The UTHealth Houston, IRB, approved this study HSC-SPH-24-0335. The IRB approved this protocol, information sheets, training curriculum, and recruitment materials. If we need to make any modifications to the protocol which may impact on the conduct of the study, a formal amendment to the protocol will be submitted to UTHealth Houston IRB for approval and the PI will communicate changes to implementation collaborators.

### Consent process

Providers, staff, and parents will complete electronic* s*urveys remotely or in person. Research staff will obtain informed consent before pre-post surveys and document consent in REDCap. For remote interviews, research staff will obtain verbal consent from interviewees via video conference and document consent in REDCap. The participants for both surveys and interviews will receive a copy of the consent form via email. See Supplementary Files 4-7.

## Discussion

Despite HPV vaccine intervention research showing that focusing on implementation on multiple levels and using theory in intervention development are best practices, few interventions were multilevel and employed theory [52]. Additionally, implementation studies to increase vaccination in resource-limited settings like FQHCs [59–61], especially those serving low-income, uninsured/underinsured, and racial/ethnic minority populations, are limited. Our study addresses these gaps by focusing on multiple levels (provider and practice) and employing frameworks (i-PARIHS and ISF). The study will provide three main outcomes: a summary report of the baseline assessments, implementation and effectiveness outcome data, and observational data. First, the summary report includes vaccine coverage, provider performance, and practice characteristics the implementation team will utilize to select specific implementation strategies to increase vaccinations and will be included in their clinical practice plan. Second, the effectiveness and implementation outcome data will provide evidence for implementation efforts directed towards increasing vaccinations for Latino/a adolescents and in the long-term will reduce health disparities associated with HPV-associated cancers. Findings will also provide information on which implementation strategies work best in resource-limited practice settings to increase recommendations and vaccinations. Lastly, the observational data from the implementation period and the interviews will collectively contribute to the limited empirical literature around HPV vaccination-related implementation strategies and outcomes. These findings will help identify factors influencing sustainability, as perceived by implementation team that were involved in the implementation and help distinguish whether they are distinct from factors influencing implementation.

We also may encounter potential challenges related to quality assurance, retention, and training delivery involved in performing the study, but have devised alternative strategies to overcome these challenges. First, if practices are not able to generate vaccination status reports from their EHR, we have experience from our pilot in troubleshooting this problem and ensuring high assessment quality through extensive training and ongoing technical assistance for practice staff (remotely or by phone). The research team also has substantial experience in collecting data in person and remotely and in both English and Spanish. Another potential problem is clinical staff turnover, which we also have experience from our pilot troubleshooting by the external facilitator working closely with FQHC leadership to implement strategies and booster trainings for new staff. These trainings ensure the capacity of the practice for sustaining their clinical practice plans. Lastly, regarding training delivery, we are working with the TACHC to tailor the delivery (in person, remote or hybrid) of the training to fit FQHC needs. TACHC has extensive experience planning, delivering, and evaluating remotely delivered trainings with FQHCs. TACHC has agreed to provide infrastructure support, guidance, and coordination for transitioning provider and immunization navigator training to a remotely delivered model and implementing monthly problem-solving meetings across practices.

In conclusion, the findings of this study will advance our understanding of implementation research in resource-limited practices that serve populations experiencing substantial disparities, and to identify strategies that improve delivery and increase vaccine uptake. Findings from the current study will inform national implementation efforts and contribute towards future research targeting dissemination and scale-up, key foci for health equity focused implementation research.

## Supplementary Information

Supplementary Material 1. SPIRIT checklist.

Supplementary Material 2. WHO Trial Registration Data.

Supplementary Material 3. Letter of Information for survey and intervention – Providers.

Supplementary Material 4. Letter of Information for survey and intervention – Staff.

Supplementary Material 5. Letter of Information for interviews – Implementation Team.

Supplementary Material 6. Letter of Information for survey – Parents (English).

Supplementary Material 7. Letter of Information for survey – Parents (Spanish).

## Data Availability

No datasets were generated or analysed during the current study.

## Abbreviations

CDC: Center for Disease Control and Prevention
CHN: Community Health Network
EHR: Electronic Health Record
FQHC: Federally Qualified Health Center
HCP: Healthcare Provider
HPV: Human papillomavirus
i-PARIHS: Integrated-Promoting Action on Research Implementation in Health Services
ISF: Integrated Sustainability Framework
MenACWY: Meningococcal A, C, W, and Y conjugate vaccine
PVHC: Project Vida Health Center
SBCHC: Spring Branch Community Health Center
TACHC: Texas Association of Community Health Centers
Task Force: Community Preventive Services Task Force
Tdap: Tetanus, diphtheria, and pertussis vaccine
TRUE SYNERGI: TRUsted hEalth SYstem implementatioN stratEGIes to increase vaccination
UTHealth Houston: The University of Texas Health Science Center at Houston
