# Supplementary material for: Trusted health system implementation strategies to increase vaccination (TRUE SYNERGI): a stepped-wedge cluster randomized trial to reduce HPV-related cancers
Source: BMC Public Health. 2025 Apr 9;25:1331. doi: 10.1186/s12889-025-22273-7 (PMC11983866; doi:10.1186/s12889-025-22273-7)
Supplement: Supplementary file 2 — Supplementary Material 2. WHO Trial Registration Data. [file 12889_2025_22273_MOESM2_ESM.docx]

| **Data category** | **Information** |
| --- | --- |
| Primary registry and trial identifying number | Clinicaltrails.gov NCT06598475 |
| Date of registration in primary registry | 13 September 2024 |
| Secondary identifying numbers | HSC-SPH-24-0335 |
| Source(s) of monetary of material support | National Cancer Institute (NCI) |
| Primary sponsor | The University of Texas Health Science Center, Houston |
| Secondary sponsor(s) | Not applicable |
| Contact for public queries | PhD, Daisy Y Morales-Campos; Email: Daisy.Y.MoralesCampos@uth.tmc.edu |
| Contact for scientific queries | PhD, Daisy Y Morales-Campos |
| Public title | Trusted Health System Implementation Strategies to increase vaccination (TRUE SYNERGI): A stepped-wedge cluster randomized trial to reduce HPV-related cancers |
| Scientific title | Investigating facilitator-driven, multi-level implementation strategies in FQHCs to improve provider recommendation and HPV vaccination rates among Latino/a adolescents |
| Countries of recruitment | United States |
| Health condition(s) or problem(s) studied | Human Papillomavirus (HPV), HPV vaccination, Cancer Prevention |
| Intervention(s) | Facilitator-driven provider- and practice-level implementation strategies: provider education, clinical practice plan, immunization navigator education, technical assistance, assessment and feedback, booster trainings |
| Key inclusion and exclusion criteria | Ages eligible for study: ≥ 18 years; Sexes eligible for study: Both; Other: Provider, staff, implementation team member employed at the FQHC; Parent of patient 11-17 seeking care at the FQHC  Inclusion Criteria: FQHC's with less than 60% HPV vaccine initiation rate for 11-12 year old adolescents overall at their practice sites; have family medicine and/or pediatric practices; and a total adolescent patient population of at least 50% Latino.  Exclusion Criteria: FQHCs that participated in the pilot study were excluded. |
| Study type | Interventional; Allocation: Randomized; Masking: Not Applicable; Assignment: Crossover; Purpose: Prevention; Phases: Not Applicable |
| Date of first enrollment | March 2025 |
| Target sample size | 2142 |
| Recruitment status | Pending: participants are not yet being recruited or enrolled at any site. |
| Primary outcome(s) | Timely HPV vaccine initiation and completion rates of 11-12 year old’s. |
| Key secondary outcomes | Timely HPV vaccine initiation and completion for adolescent patients 13-17 years old; Percentage of non-well visits in which HPV vaccine was not administered, among all visits for active vaccine-eligible patients ages 11-17 by clinic. Percentage of adolescent patients at each clinic who received Tdap and MenACWY at age 11. |
