# Supplementary material for: Trusted health system implementation strategies to increase vaccination (TRUE SYNERGI): a stepped-wedge cluster randomized trial to reduce HPV-related cancers
Source: BMC Public Health. 2025 Apr 9;25:1331. doi: 10.1186/s12889-025-22273-7 (PMC11983866; doi:10.1186/s12889-025-22273-7)
Supplement: Supplementary file 3 — Supplementary Material 3. Letter of Information for survey and intervention – Providers. [file 12889_2025_22273_MOESM3_ESM.pdf]

LETTER OF INFORMATION
TO TAKE PART IN RESEARCH

Study Title: Investigating facilitator-driven, multi-level implementation strategies in Federally Qualified Health Centers to improve provider recommendation and HPV vaccination rates among Latino/a adolescents

Study Sponsor: NIH National Cancer Institute

Principal Investigator: Daisy Y. Morales-Campos, PhD, Associate Professor, School of Public Health, UTHealth Houston

IRB Number: HSC-SPH-24-0335

First, the purpose of the surveys is to assess provider's HPV vaccine knowledge, attitudes, confidence responding to vaccine hesitant parents (general and HPV related), and both provider-and practice-level behaviors regarding HPV vaccination (i.e., current vaccine administration, frequency of administration and immunization history). You are invited to take part in this study because you are a provider at one of the study practice sites. We will ask 4 providers per practice to take surveys three months before project activities start and 12 months after project activities start and receive an intervention as part of the project activities. Second, the purpose of the intervention is to educate health care providers on evidence-based HPV vaccination practices and utilize the new knowledge and skills to both improve clinical practice and increase vaccination rates.

If you agree to participate, you will be asked to:
Surveys:

- We will invite providers at baseline (three months before project activities start) and post assessment (12 months after project activities start) periods to participate in the anonymous electronic surveys.
- We will ask you to fill out a survey describing your HPV vaccine knowledge, attitudes, confidence responding to vaccine hesitant parents, and both provider-and practice-level behaviors regarding HPV vaccination.
- The survey will take 15-20 minutes to complete, and you will not put your name on it.

Intervention:

- Providers will receive brief, facilitator-led, either remotely delivered or face-to-face in their offices evidence-based education sessions as part of the project activities. The facilitator can tailor the length (5, 10, or 20 minutes) of the session according to providers' availability.
- The content for sessions consists of:

Content	
Session 1	Understanding the Burden of HPV infection & Disease
Session 2	Evidence – Based HPV disease prevention
Session 3	Talking about HPV - framing the conversation
Session 4	Strategies to improve HPV vaccine coverage
Session 5-6	Development of practice plans to increase vaccination rates

- The facilitator will schedule sessions according to the providers' availability.
- The facilitator will do the following with providers:

Conduct Sessions	Provide CME Credits	Provide Assessment and Feedback
• **Quantity**: 4 education sessions, plus 2 visits to create a practice plan<br>• **Length:** Each session can be tailored in length (5, 10, or 20 minutes) according to providers' availability	Providers will receive:<br>• 4 CME credits (1 per completion of each session and evaluation survey).<br>• 2 CME credits for creating a clinic practice plan with the assistance of the facilitator.<br>• A certification of completion.	• Report every three months to providers on rates of HPV vaccinations delivered<br>• Report how providers' practice plans are functioning in their practice

The risks to participating in this study are minimal (i.e., loss of confidentiality) but no greater than those encountered in everyday life. You may not receive any benefits from participating in this study. Although you may not receive a personal benefit from participating, we hope the lessons we learn will benefit this community health center and its patients by improving HPV vaccination initiation and completion rates for adolescents and improve clinical practice.

There are no costs to you, and you will not be paid to participate in this study, but participants in the Clinic Member Survey will be entered into a drawing for a tablet. The UTHealth Science Center in Houston research staff will conduct a single drawing for the baseline period and a single drawing during the post-assessment period. One participant will be selected in the baseline and post-assessment period and notified by email to provide information on where to pick up the tablet. The odds of selecting a single participant as a result of the drawing will be one in four. You can refuse to answer any questions asked or written on any form. Your participation in this study is voluntary. A decision not to participate in this study will not change the services available to you from the PI or study staff.

We will protect any information we collect from you by doing the following:

- Any personal information that you provide will be kept confidential to every extent of the law.
- We will not identify you if we publish interview results in a report, presentation, journal, or book.
- Your name will not appear on any interview documents or audio files. All written and electronic forms and study materials will be kept secure. Your response(s) to questions may appear as de-identified quotes, so anything that could identify you or anyone you refer to will be removed. All written materials will be stored in a locked file in the program's office.
- We will share deidentified data with other researchers once the study ends.
- Information about you may be given to the study sponsor and/or representative of the sponsor and the UTHealth Houston Institutional Review Board and our study collaborators at the University of Texas at Austin, the University of Maryland, the University of New Mexico, and Albert Einstein College of Medicine.
- A description of this study will be available on http://www.ClinicalTrials.gov as required by U.S. law. This web site will not include information that can identify you. At most, the web site will include a summary of the results. You can search this web site at any time.
- To help us protect your privacy we have obtained a Certificate of Confidentiality from the National Institutes of Health. With this Certificate, the researchers cannot be forced to disclose information that may identify you, even by a court subpoena, in any federal, state, or local civil, criminal, administrative, legislative, or other proceedings. The researchers will use the certificate to resist any demands for information that would identify you, except as explained below. The certificate cannot be used to resist a demand for information from personnel of the United States Government that is used for auditing or evaluation of federally funded projects or for information that must be disclosed to meet the requirements of the federal Food and Drug Administration (FDA). A Certificate of Confidentiality does not prevent you or a member of your family from voluntarily releasing information about yourself or your involvement in this research. If an insurer, employer, or other person obtains your written consent to receive research information, then the researchers may not use the Certificate to withhold that information.

If you have any questions about this project, please contact research coordinator at (713) 500-9654.

This research project has been reviewed by the Committee for the Protection of Human Subjects (CPHS) of the University of Texas Health Science Center at Houston, as HSC-SPH-24-0335. For any questions about your rights as a research subject, please call CPHS at (713) 500-7943.

This form is yours to keep.
