# Supplementary material for: Trusted health system implementation strategies to increase vaccination (TRUE SYNERGI): a stepped-wedge cluster randomized trial to reduce HPV-related cancers
Source: BMC Public Health. 2025 Apr 9;25:1331. doi: 10.1186/s12889-025-22273-7 (PMC11983866; doi:10.1186/s12889-025-22273-7)
Supplement: Supplementary file 4 — Supplementary Material 4. Letter of Information for survey and intervention – Staff. [file 12889_2025_22273_MOESM4_ESM.pdf]

LETTER OF INFORMATION
TO TAKE PART IN RESEARCH

Study Title: Investigating facilitator-driven, multi-level implementation strategies in Federally Qualified Health Centers to improve provider recommendation and HPV vaccination rates among Latino/a adolescents

Study Sponsor: NIH National Cancer Institute

Principal Investigator: Daisy Y. Morales-Campos, PhD, Associate Professor, School of Public Health, UTHealth Houston

IRB Number: HSC-SPH-24-0335

First, the purpose of the surveys is to assess staff's HPV vaccine knowledge, attitudes, confidence responding to vaccine hesitant parents (general and HPV related), and both provider-and practice level behaviors regarding HPV vaccination (i.e., current vaccine administration, frequency of administration and immunization history). You are invited to take part in this study because you are staff at one of the study practice sites. We will ask 5 staff per practice to take surveys three months before project activities start and 12 months after project activities start. Second, the purpose of the intervention is to educate staff selected by administrators to be immunization navigators on evidence-based HPV vaccination practices and utilize the new knowledge and skills to both improve clinical practice and increase vaccination rates. You are invited to take part in this study because you are staff at one of the study practice sites. Practice administrators will select 3 staff per practice to be immunization navigators and receive an intervention as part of the project activities.

If you agree to participate, you will be asked to:
Surveys:

- We will invite staff at baseline (three months before project activities start) and post assessment (12 months after project activities start) periods to participate in the anonymous electronic surveys.
- We will ask you to fill out a survey describing your HPV vaccine knowledge, attitudes, confidence responding to vaccine hesitant parents, and both provider-and practice-level behaviors regarding HPV vaccination.
- The survey will take 15-20 minutes to complete, and you will not put your name on it.

Intervention:

- Staff selected to be immunization navigators by administrators will receive facilitator-led, either remotely delivered or face-to-face evidence-based education sessions. The content and length for sessions is listed below:

Content	
Session 1	Immunization 101 (90 minutes)
Session 2	Utilizing evidenced-based intervention to improve vaccination rates (115 minutes)
Session 3	Vaccination storage and handling (20 minutes)

- The facilitator will schedule sessions according to the staffs' availability.
- The facilitator will train immunization navigators to implement the practice plan developed by the provider for each practice and provide ongoing technical assistance.
- The facilitator will conduct booster trainings and site visits every six months, to observe and provide technical assistance and corrective feedback to ensure that performance does not deteriorate over time.

The risks to participating in this study are minimal but no greater than those encountered in everyday life. You may not receive any benefits from participating in this study. Although you may not receive a personal benefit from participating, we hope the lessons we learn will benefit this community health center and its patients by improving HPV vaccination initiation and completion rates for adolescents and improve clinical practice.

There are no costs to you, and you will not be paid to participate in this study, but participants in the Clinic Member Survey will be entered into a drawing for a tablet. The UTHealth Science Center in Houston research staff will conduct a single drawing for the baseline period and a single drawing during the post-assessment period. One participant will be selected in the baseline and post-assessment period and notified by email to provide information on where to pick up the tablet. The odds of selecting a single participant as a result of the drawing will be one in five. You can refuse to answer any questions asked or written on any form. Your participation in this study is voluntary. A decision not to participate in this study will not change the services available to you from the PI or study staff.

We will protect any information we collect from you by doing the following:

- Any personal information that you provide will be kept confidential to every extent of the law.
- We will not identify you if we publish interview results in a report, presentation, journal, or book.
- Your name will not appear on any interview documents or audio files. All written and electronic forms and study materials will be kept secure. Your response(s) to questions may appear as de-identified quotes, so anything that could identify you or anyone you refer to will be removed. All written materials will be stored in a locked file in the program's office.
- We will share deidentified data with other researchers once the study ends.
- Information about you may be given to the study sponsor and/or representative of the sponsor and the UTHealth Houston Institutional Review Board and our study collaborators at the University of Texas at Austin, the University of Maryland, the University of New Mexico, and Albert Einstein College of Medicine.

- A description of this study will be available on http://www.ClinicalTrials.gov as required by U.S. law. This web site will not include information that can identify you. At most, the web site will include a summary of the results. You can search this web site at any time.
- To help us protect your privacy we have obtained a Certificate of Confidentiality from the National Institutes of Health. With this Certificate, the researchers cannot be forced to disclose information that may identify you, even by a court subpoena, in any federal, state, or local civil, criminal, administrative, legislative, or other proceedings. The researchers will use the certificate to resist any demands for information that would identify you, except as explained below. The certificate cannot be used to resist a demand for information from personnel of the United States Government that is used for auditing or evaluation of federally funded projects or for information that must be disclosed to meet the requirements of the federal Food and Drug Administration (FDA). A Certificate of Confidentiality does not prevent you or a member of your family from voluntarily releasing information about yourself or your involvement in this research. If an insurer, employer, or other person obtains your written consent to receive research information, then the researchers may not use the Certificate to withhold that information.

If you have any questions about this project, please contact research coordinator at (713) 500-9654.

This research project has been reviewed by the Committee for the Protection of Human Subjects (CPHS) of the University of Texas Health Science Center at Houston, HSC-SPH-24-0335. For any questions about your rights as a research subject, please call CPHS at (713) 500-7943.

This form is yours to keep.
