# Supplementary material for: Trusted health system implementation strategies to increase vaccination (TRUE SYNERGI): a stepped-wedge cluster randomized trial to reduce HPV-related cancers
Source: BMC Public Health. 2025 Apr 9;25:1331. doi: 10.1186/s12889-025-22273-7 (PMC11983866; doi:10.1186/s12889-025-22273-7)
Supplement: Supplementary file 5 — Supplementary Material 5. Letter of Information for interviews – Implementation Team. [file 12889_2025_22273_MOESM5_ESM.pdf]

**LETTER OF INFORMATION**
**TO TAKE PART IN RESEARCH**

**Study Title:** Investigating facilitator-driven, multi-level implementation strategies in Federally Qualified Health Centers to improve provider recommendation and HPV vaccination rates among Latino/a adolescents

**Study Sponsor:** NIH National Cancer Institute

**Principal Investigator:** Daisy Y. Morales-Campos, PhD, Associate Professor, School of Public Health, UTHealth Houston

**IRB Number:** HSC-SPH-24-0335

The purpose of the implementation team interviews is to conduct a practice readiness assessment, facilitate a discussion around your practice level data, and identify challenges to achieving target vaccination rates. You are invited to take part in this study because you comprise the practice implementation team (i.e., administrative designee, health care provider, immunization navigator, or other staff). We will ask four members per practice in the study to participate.

If you agree to participate, you will be asked to:

1. Fill out a short survey describing yourself (your age, gender, race/ethnicity, language spoken, country of birth, years living in the United States and Texas) and your practice prior to Interview #1. The form will take 5 minutes to complete, and you will not put your name on it.
2. After you complete the survey, you will take part in Interview #1 that should last about 3060 minutes and will be audio recorded so that we will have an accurate record of what you say. The audio recordings of the interview will be transferred into paper format (by listening to the audio recordings and typing word for word what participants said). We will keep the audio files on file for no longer than 12 months and then they will be destroyed.
3. Twelve months following the implementation of program activities, we will invite you to participate in Interview #2 to identify key factors that influence sustainability of the strategies employed in your practice. We will use these interviews to gain in-depth insight and explore the real-world complexity associated with sustaining interventions in FQHCs. Interview #2 should last about 30-60 minutes and will be audio recorded so that we will have an accurate record of what you say. The audio recordings of the interview will be transferred into paper format (by listening to the audio recordings and typing word for word what participants said). We will keep the audio files on file for no longer than 12 months and then they will be destroyed.

The risks to participating in this study are minimal (i.e., loss of confidentiality) but no greater than those encountered in everyday life. You may not receive any benefits from participating in this study, we hope the lessons we learn will benefit this community health center and its patients.

There are no costs to you, and you will not be paid to take part in this study. You will not be personally identified in any reports or publications that may result from this study. Any personal information about you that is gathered during this study will remain confidential to the extent of the law. You can refuse to answer any questions asked or written on any forms. Your participation in this study is voluntary. A decision not to take part in this study will not change the services available to you from the PI or study staff.

We will protect any information we collect from you by doing the following:

- Any personal information that you provide will always be kept confidential to every extent possible.
- We will not identify you if we publish interview results in a report, presentation, journal, or book.
- Your name will not appear on any interview documents or audio files. All written and electronic forms and study materials will be kept secure. Your response(s) to questions may appear as de-identified quotes, so anything that could identify you or anyone you refer to will be removed. All written materials will be stored in a locked file in the program's office.
- We will share deidentified data with other researchers once the study ends.
- Information about you may be given to the study sponsor and/or representative of the sponsor and the UTHealth Houston Institutional Review Board and our study collaborators at the University of Texas at Austin, the University of Maryland, the University of New Mexico, and Albert Einstein College of Medicine.
- A description of this study will be available on http://www.ClinicalTrials.gov as required by U.S. law. This web site will not include information that can identify you. At most, the web site will include a summary of the results. You can search this web site at any time.
- To help us protect your privacy we have obtained a Certificate of Confidentiality from the National Institutes of Health. With this Certificate, the researchers cannot be forced to disclose information that may identify you, even by a court subpoena, in any federal, state, or local civil, criminal, administrative, legislative, or other proceedings. The researchers will use the certificate to resist any demands for information that would identify you, except as explained below. The certificate cannot be used to resist a demand for information from personnel of the United States Government that is used for auditing or evaluation of federally funded projects or for information that must be disclosed to meet the requirements of the federal Food and Drug Administration (FDA). A Certificate of Confidentiality does not prevent you or a member of your family from voluntarily releasing information about yourself or your involvement in this research. If an insurer, employer, or other person obtains your written consent to receive research information, then the researchers may not use the Certificate to withhold that information.

If you have any questions about this project, please contact project coordinator at (713) 500-9654.

This research project has been reviewed by the Committee for the Protection of Human Subjects (CPHS) of the University of Texas Health Science Center at Houston, HSC-SPH-24-0335. For any questions about your rights as a research subject, please call CPHS at (713) 500-7943.

This form is yours to keep.
