# Supplementary material for: Trusted health system implementation strategies to increase vaccination (TRUE SYNERGI): a stepped-wedge cluster randomized trial to reduce HPV-related cancers
Source: BMC Public Health. 2025 Apr 9;25:1331. doi: 10.1186/s12889-025-22273-7 (PMC11983866; doi:10.1186/s12889-025-22273-7)
Supplement: Supplementary file 6 — Supplementary Material 6. Letter of Information for survey – Parents (English). [file 12889_2025_22273_MOESM6_ESM.pdf]

LETTER OF INFORMATION
TO TAKE PART IN RESEARCH

Study Title: Investigating facilitator-driven, multi-level implementation strategies in Federally Qualified Health Centers to improve provider recommendation and HPV vaccination rates among Latino/a adolescents

Study Sponsor: NIH National Cancer Institute

Principal Investigator: Daisy Y. Morales-Campos, PhD, Associate Professor, School of Public Health, UTHealth Houston

IRB Number: HSC-SPH-24-0335

The purpose of the surveys is to evaluate parents' human papillomavirus (HPV) vaccine knowledge, behaviors, concerns related to vaccine side effects and doses, self-efficacy in and reducing perceived barriers to vaccinating their child, and experience with provider's HPV vaccine recommendation and satisfaction with practice services. You are invited to take part in this study because you are a parent of a patient ages 11-17 seeking care at the practice. We will ask 25 parents at each practice to participate three months before practice activities start and 25 parents at each practice to participate 12 months after practice activities start.

Practice staff will invite parents of patients ages 11–17 who are scheduled for well-child check during the baseline (three months before project activities start) and post assessment (12 months after project activities start) periods and have not received the HPV vaccine to participate in the anonymous electronic surveys. If you agree to participate, you will be asked to fill out a survey describing your HPV vaccine knowledge, behaviors, concerns related to vaccine side effects and doses, self-efficacy in and reducing perceived barriers to vaccinating their child, and experience with provider's HPV vaccine recommendation and satisfaction with practice services. The survey will take 15-20 minutes to complete, and you will not put your name on it.

The risks to participating in this study are minimal (i.e., loss of confidentiality) but no greater than those encountered in everyday life. You may not receive any benefits from participating in this study. Although you may not receive a personal benefit from participating, we hope the lessons we learn will benefit this community health center and its patients.

There are no costs associated with participation in this study, and participants will not be compensated in monetary form for their participation. However, eligible participants will receive a $10 Walmart gift card as a token of appreciation. Participation is entirely voluntary, and you may refuse to answer any questions or skip any items on forms without penalty. Your decision to participate will not impact the services available to you from the Principal Investigator (PI) or study staff.

We will protect any information we collect from you by doing the following:

- Any personal information that you provide will be kept confidential to every extent of the law.
- We will not identify you if we publish interview results in a report, presentation, journal, or book.
- Your name will not appear on any interview documents or audio files. All written and electronic forms and study materials will be kept secure. Your response(s) to questions may appear as de-identified quotes, so anything that could identify you or anyone you refer to will be removed. All written materials will be stored in a locked file in the program's office.
- We will share deidentified data with other researchers once the study ends.
- Information about you may be given to the study sponsor and/or representative of the sponsor and the University of Texas at Austin Institutional Review Board and our study collaborators at the University of Maryland, the University of New Mexico, and Albert Einstein College of Medicine.
- A description of this study will be available on http://www.ClinicalTrials.gov as required by U.S. law. This web site will not include information that can identify you. At most, the web site will include a summary of the results. You can search this web site at any time.
- To help us protect your privacy we have obtained a Certificate of Confidentiality from the National Institutes of Health. With this Certificate, the researchers cannot be forced to disclose information that may identify you, even by a court subpoena, in any federal, state, or local civil, criminal, administrative, legislative, or other proceedings. The researchers will use the certificate to resist any demands for information that would identify you, except as explained below. The certificate cannot be used to resist a demand for information from personnel of the United States Government that is used for auditing or evaluation of federally funded projects or for information that must be disclosed to meet the requirements of the federal Food and Drug Administration (FDA). A Certificate of Confidentiality does not prevent you or a member of your family from voluntarily releasing information about yourself or your involvement in this research. If an insurer, employer, or other person obtains your written consent to receive research information, then the researchers may not use the Certificate to withhold that information.

If you have any questions about this project, please contact project coordinator at (713) 500-9654.

This research project has been reviewed by the Committee for the Protection of Human Subjects (CPHS) of the University of Texas Health Science Center at Houston, as HSC-SPH-24-0335. For any questions about your rights as a research subject, please call CPHS at (713) 500-7943.

This form is yours to keep.
