# Supplementary material for: Trusted health system implementation strategies to increase vaccination (TRUE SYNERGI): a stepped-wedge cluster randomized trial to reduce HPV-related cancers
Source: BMC Public Health. 2025 Apr 9;25:1331. doi: 10.1186/s12889-025-22273-7 (PMC11983866; doi:10.1186/s12889-025-22273-7)
Supplement: Supplementary file 7 — Supplementary Material 7. Letter of Information for survey – Parents (Spanish). [file 12889_2025_22273_MOESM7_ESM.pdf]

EVALUACION DE REFERENCIA
FORMULARIO DE CONSENTIMIENTO PARA ENCUESTAS PARA/DE PADRES

Titulo: Investigando estrategias de implementación de múltiples niveles impulsadas por facilitadores en Centros de Salud Federalmente Calificados para mejorar la recomendación de los proveedores y las tasas de vacunación contra el VPH entre los latinos adolescentes

Se le pide que particIpe en un estudio de investigación. Este formulario le brinda información importante sobre las encuestas. Lea la información a continuación y haga cualquier pregunta que pueda tener antes de decider si participar.

¿Quién está llevando a cabo este proyecto y cuál es el propósito de las encuestas?

- Daisy Y. Morales-Campos, PhD, Profesor Asistente de Investigación, Universidad de Salud Publica en UTHealth Houston.
- El Instituto Nacional del Cáncer es el patrocinador del estudio.
- El propósito de las encuestas es evaluar el conocimiento de los padres sobre la vacuna contra el virus del papilloma humano (VPH), los comportamientos, las preocupaciones relacionadas con los efectos secundarios y las dosis de la vacuna, la autoeficacia y la reducción de las barreras percibidas para vacunar a sus hijos, y la experiencia con la recomendación de la vacuna contra el VPH del proveedor y satisfacción con los servicios de práctica.

¿A quién se le pide que participe?

- 25 padres de pacientes de 11 a 17 años de edad que buscan atención en cada consultorio tres meses antes de que comiencen las actividades del proyecto.
- 25 padres de pacientes de 11 a 17 años de edad que buscan atención en cada consultorio 12 meses después del inicio de las actividades del proyecto.

¿Tiene que participar?

- Su participación en las encuestas es completamente voluntaria.
- Usted es libre de retirarse en cualquier momento indicando que ya no desea participar en las encuestas. Cualquier dato personal recopilado no se incluiará en el análisis y será destruido.

Procedimientos

- El personal de práctica invitará a los padres de pacientes de 11 a 17 años de edad que tengan programado un control de niño sano durante los períodos inicial (tres meses antes de que comiencen las actividades del proyecto) y posterior a la evaluación (12 meses después del inicio de las actividades del proyecto) y que no hayan recibido la vacuna contra el VPH para participar en las encuestas electrónicas anónimas.
- Le pediremos que complete una encuesta que describa sus conocimientos sobre la vacuna contra el VPH, sus comportamientos, sus inquietudes relacionadas con los efectos secundarios y las dosis de

la vacuna, su autoeficacia y la reducción de las barreras percibidas para vacunar a su hijo/a, y su experiencia con la recomendación de la vacuna contra el VPH del proveedor y su satisfacción con servicios de práctica.

- La encuesta tardará entre 15 y 20 minutos en completarse y no pondrá su nombre en ella.

Riesgos y Beneficios

- Existen riesgos mínimos (es decir, pérdida de confidencialidad) asociados con su participación en estas encuestas, pero no mayores que los que se encuentran en la vida cotidiana. No hay beneficios directos para usted como participante en estas encuestas.
- Aunque es posible que no reciba un beneficio personal al participar, esperamos que las lecciones que aprendamos beneficien a este centro de salud comunitario y a sus pacientes.

Compensación

- Participar en este estudio no tiene ningún costo y los participantes no recibirán compensación económica por su participación. Sin embargo, los participantes que cumplan los requisitos recibirán una tarjeta de de Walmart de $10 como muestra de agradecimiento. La participación es totalmente voluntaria y puede negarse a responder preguntas o omitir cualquier elemento de los formularios sin penalización. Su decisión de participar no afectará los servicios que le ofrecen el Investigador Principal (IP) ni el personal del estudio.

¿Cómo protegeremos su información?

Protegeremos cualquier información que recopilemos de usted hacienda lo siguiente:

- Cualquier información personal que usted proporcione siempre se mantendrá confidencial en la medida de lo posible.
- No lo identificaremos si publicamos los resultados de la entrevista en un informe, presentación, revista o libro.
- Su nombre no aparecerá en ningún documento de entrevista o archive de audio. Todos los formularios escritos y electrónicos y materiales de estudio se mantendrán seguros. Sus respuestas a las preguntas pueden aparecer como citas no identificadas, por lo que se eliminará cualquier cosa que pueda identificarlo a usted o a cualquier persona a la que se refiera. Todos los materiales escritos se almacenarán en un archive bajo llave en la oficina del programa.
- Compartiremos datos no identificados con otros investigadores una vez que finalice el estudio.
- Se puede proporcionar información sobre usted al patrocinador del estudio y/o al representante del patrocinador y a la Junta de Revisión Institucional de la Universidad de UTHealth en Houston y a nuestros colaboradores el estudio en la Universidad de Texas en Austin, Universidad de Maryland, Universidad de Nuevo México y Universidad Albert Einstein Collegio de Medicina.
- Una descripción de este estudio estará disponible en http://www.ClinicalTrials.gov. Este sitio web no incluirá información que pueda identificarlo. Como máximo, el sitio Web incluirá un resumen de los resultados. Puede buscar en este sitio web en cualquier momento

- Para ayudarnos a proteger su privacidad, hemos obtenido un Certificado de Confidencialidad de los Institutos Nacionales de Salud. Con este Certificado, los investigadores no pueden ser obligados a revelar información que pueda identificarlo, incluso mediante una citación judicial, en ningún procedimiento civil, penal, administrative, legislativo o de otro tipo federal, estatal o local. Los investigadores utilizarán el certificado para resistir cualquier demanda de información que pueda identificarlo, excepto como se explica a continuación. El certificado no se puede utilizar para resistir una demanda de información del personal del Gobierno de los Estados Unidos que se utiliza para auditor o evaluar proyectos financiados con fondos federales o para información que debe divulgarse para cumplir con los requisites de la Administración de Alimentos y Medicamentos (FDA) federal. Un Certificado de Confidencialidad no impide que usted o un miembro de su familia divulgue voluntariamente información sobre usted o su participación en este investigación. Si una aseguradora, empleador u otra persona obtiene su consentimiento por escrito para recibir información de investigación, entonces los investigadores no podrán usar el Certificado para retener esa información.

Información de contacto del equipo de estudio

- Si tiene alguna pregunta o inquietud, comuníquese con: Coordinador del programa Patricia Ramirez al (713) 500-9654.

Información de contacto para preguntas sobre sus derechos como participante de la investigación

Si tiene preguntas sobre sus derechos como participante de una investigación, o desea obtener información, hacer preguntas o discutir cualquier inquietud sobre este estudio con alguien que no sea el investigador, comuníquese con lo siguiente:

La Junta de Revisión Institucional de la Universidad de Texas Health Science Center en Houston, HSC-SPH-24-0335 al numero (713) 500-7943.

Este formulario es suyo.
